# Modern antiseptics against multidrug-resistant *Pseudomonas aeruginosa*, emerging from war-related injuries in Ukraine

**DOI:** 10.3389/fmicb.2025.1656270

**Published:** 2025-10-31

**Authors:** Oleksandr Nazarchuk, Kristian Riesbeck, Valentyn Kovalchuk, Tetiana Denysko, Mariia Faustova, Roman Chornopyshchuk, Halyna Nazarchuk, Oleg Parkhomenko, Nataliia Bahniuk, Dmytro Dmytriiev, Vasyl Nagaichuk

**Affiliations:** ^1^Department of Microbiology, National Pirogov Memorial Medical University, Vinnytsia, Ukraine; ^2^Center of Thermal Injury and Plastic Surgery, MNPE “Vinnytsia Regional Clinical Hospital Vinnytsia Regional Council”, Vinnytsia, Ukraine; ^3^Clinical Microbiology, Department of Translational Medicine, Faculty of Medicine, Lund University, Malmö, Sweden; ^4^Department of General and Clinical Epidemiology and Biosafety with a Course on Microbiology and Virology, Odesa National Medical University, Odesa, Ukraine; ^5^Department of Microbiology, Virology and Immunology, Poltava State Medical University, Poltava, Ukraine; ^6^Department of General Surgery, National Pirogov Memorial Medical University, Vinnytsya, Ukraine; ^7^Department of Ophthalmology, National Pirogov Memorial Medical University, Vinnytsya, Ukraine; ^8^Central Policlinic of Internal Affairs of Ukraine, Kyiv, Ukraine; ^9^Department of Anesthesiology, Intensive Care and Emergency Medicine, National Pirogov Memorial Medical University, Vinnytsya, Ukraine

**Keywords:** multidrug-resistant bacteria, *Pseudomonas aeruginosa*, healthcare-associated infections, antiseptics, susceptibility to antiseptics, quaternary ammonium compounds, biguanide compounds, anti-biofilm activity

## Abstract

Susceptibility testing of clinical multidrug-resistant (MDR) and reference *P. aeruginosa* strains was performed using the standard twofold serial dilution method. The minimum inhibitory concentration (MIC) and minimum bactericidal concentration (MBC) of antiseptics were determined. MIC and MBC values were also interpreted as the bacteriostatic index of antiseptic activity (BSIAA) and the bactericidal index of antiseptic activity (BCIAA). The ability of strains to form biofilms, the inhibition of biofilm formation, and the destruction of mature biofilms under the influence of bacteriostatic, bactericidal, and ½ of the initial antiseptic concentration were modeled using Christensen’s test. Antiseptics from the detergent group, decamethoxine (0.1 and 0.02%) and polyhexanide (0.1%), demonstrated the highest antimicrobial activity. Their bacteriostatic concentrations were 63.2 ± 5.2 μg/mL and 68.7 ± 4.2 μg/mL, respectively. The ranking of antiseptics by bacteriostatic efficacy was: decamethoxine > polyhexanide > octenidine > miramistin > chlorhexidine. The highest BSIAA values were observed for povidone-iodine 10%, decamethoxine 0.1%, octenidine 0.1%, and polyhexanide 0.1%. The highest bactericidal IAA values were found for povidone-iodine 10%, decamethoxine 0.1%, octenidine 0.1%, and polyhexanide 0.1%. Miramistin 0.01% was deemed insufficiently effective. Polyhexanide exhibited the highest bactericidal activity, with a BCIAA to BSIAA ratio of 0.88. For all other antiseptics, this ratio ranged from 0.5 to 0.6. All tested strains exhibited a high capacity for biofilm formation. All antiseptics significantly inhibited biofilm formation. Octenidine had the strongest effect on immature biofilms, reducing their formation by 28.5% (*p* < 0.0001). The MICs of most antiseptics stimulated mature biofilm development. The bacteriostatic concentration of octenidine led to the eradication of biofilm by 4.7% (*p* < 0.001) compared to the control. The MBC of most antiseptics (except chlorhexidine) eradicated mature biofilms by 4–30.6%, whereas chlorhexidine stimulated mature biofilm growth by 17.9%. All antiseptics, at half their initial concentration, partially eradicated MDR *Pseudomonas* biofilms by 11.3–42.4%. Analysing the effect of octenidine at different concentrations and stages of biofilm formation highlights its strong activity against *P. aeruginosa* biofilms. Our findings underscore the importance of carefully monitoring *P. aeruginosa* isolates for antiseptic susceptibility. This approach can help prevent the development of selective conditions that promote resistant microorganisms and limit their spread.

## 1 Introduction

Bacteria with multidrug resistance (MDR) have become a serious threat in the clinic. This is especially true for opportunistic pathogens, which have high natural (intrinsic) resistance, and isolates with rapidly acquired MDR. *Pseudomonas aeruginosa*, a gram-negative, rod-shaped microbe, is one of the predominant pathogens in healthcare-associated infections due to its biological flexibility and can be considered a prime example of adaptability among opportunistic pathogens (Moradali et al., 2017; Sathe et al., 2023; Sanya et al., 2023; Theuretzbacher et al., 2020; Oliver et al., 2015; Pang et al., 2019; Brüggemann et al., 2018; Murray et al., 2015; Ruffin and Brochiero, 2019).

*P. aeruginosa* is an unpretentious, versatile, and ubiquitous microbe. It can literally survive without food, not only persisting but also multiplying in distilled water due to minimal contamination. It is omnivorous, with even antimicrobial drugs serving as a source of nutrients. It can grow and multiply within a wide temperature range, from 4 to 42 °C. Being oxidase-positive, it uses oxygen as an electron acceptor but can also grow and reproduce in the absence of oxygen, where nitrate serves as the final electron acceptor, or it retains the ability to microaerobically respire (Moradali et al., 2017; Diggle and Whiteley, 2020; Favero et al., 1971; Liao et al., 2022; Nolan and Behrends, 2021).

Its “basic settings” are perfect and universal, but its initial strategy of existence and survival is not so aggressive toward humans: *P. aeruginosa* is a saprophyte that lives freely in water, soil, and can be part of the human and animal microbiome. This is true as long as we have the immune status of a healthy person. As soon as *P. aeruginosa* is able to colonize a niche, its adaptive base allows it to implement a huge range of virulence factors, even showing contact-dependent secretion of toxins directly into target cells through the type 3 secretion system (Moradali et al., 2017; Sanya et al., 2023; Diggle and Whiteley, 2020; Liao et al., 2022; Nolan and Behrends, 2021; Dasgupta et al., 2006; Goldberg et al., 2022; Balasubramanian et al., 2013).

As an opportunistic pathogen, it is also a universal pathogen, as it is pathogenic to humans, vertebrates and invertebrates, and phytopathogenic. The epidemiologically significant reservoir of hospital-acquired blue blood cell infection is medical and service personnel and patients themselves (Nolan and Behrends, 2021).

Infections caused by *P. aeruginosa* are very difficult to treat, as this microbe uses resistance mechanisms (intrinsic and acquired), forms and states of existence (planktonic and biofilm forms, persistent cell), which usually cause and lead to difficult-to-treat chronic infections (Sanya et al., 2023; Theuretzbacher et al., 2020; Driscoll et al., 2007; Lewenza et al., 2018).

A saprophytic bacterium has turned into a clinical nightmare. Its resistance and minimal nutritional requirements determine the nearly universal presence of *P. aeruginosa* in hospital environments, creating ample opportunities for the emergence of nosocomial strains. Biofilm formation is also a key factor in the success of *P. aeruginosa* as a healthcare-associated pathogen. This is especially relevant in infections of the skin and soft tissues (Thuenauer et al., 2020; Behzadi et al., 2021; Kaiser et al., 2017; Rossi Gonçalves et al., 2017; Liew et al., 2019; Ruffin and Brochiero, 2019).

The situation has become significantly more complicated in Ukraine since the start of the full-scale war. The prevalence of multidrug-resistant Gram-negative bacteria is high among those with war-related injuries (Loban’ et al., 2023). War causes special injuries, including complex fractures with bone fragmentation, traumatic limb amputations, extensive deep burns, and severe soft tissue lacerations from artillery shells and mines. The situation is complicated by the rapid infection of wounds with explosive metabolites, dirt, and dust (Loban’ et al., 2023; Melwani, 2022). It is obvious that under such conditions, the patient’s life is the main priority on the front line, which requires the immediate use of antibacterial drugs without any testing. Until the wounded arrive at specialized medical facilities, medical care is provided directly in the combat zone and at all stages of temporary evacuation, which sometimes takes days and even weeks. In addition, throughout the entire evacuation chain, additional colonization of wounds by microorganisms, very often resistant to antibiotics, occurs. This forms a special group of resistant strains, characteristic specifically of war wound infections (Loban’ et al., 2023).

Infections caused by *P. aeruginosa* require special empirical and targeted antibiotic regimens, given its innate resistance to many classes of drugs and the ability to rapidly acquire resistance to current treatments (Karruli et al., 2023). And the treatment of biofilm infections is a serious problem, as there is currently no targeted therapy that can completely destroy biofilms *in vivo* (Sathe et al., 2023; Sanya et al., 2023; Kaiser et al., 2017; Rossi Gonçalves et al., 2017).

To overcome the growing problem of resistance and taking into account the biofilm status of the pathogen, an approach involving the use of a combination of antibiotics with alternative therapies will be necessary. Combination therapy has significant advantages over conventional antibiotic therapy, as the former exerts minimal selective pressure on *P. aeruginosa* and is therefore less likely to cause drug resistance. In wound care, comprehensive topical treatment is extremely important, with antiseptic agents being a key element (Liao et al., 2022; Goldberg et al., 2022; Kramer et al., 2018; Babalska et al., 2021; Murray et al., 2015; Schultz et al., 2017; Alves et al., 2023).

This article focuses on the activity of antiseptics as important means of combating *P. aeruginosa* MDR, their ability to effectively counteract the formation of biofilm and promote its eradication.

## 2 Materials and methods

### 2.1 Study design and participants

**Figure 1:**
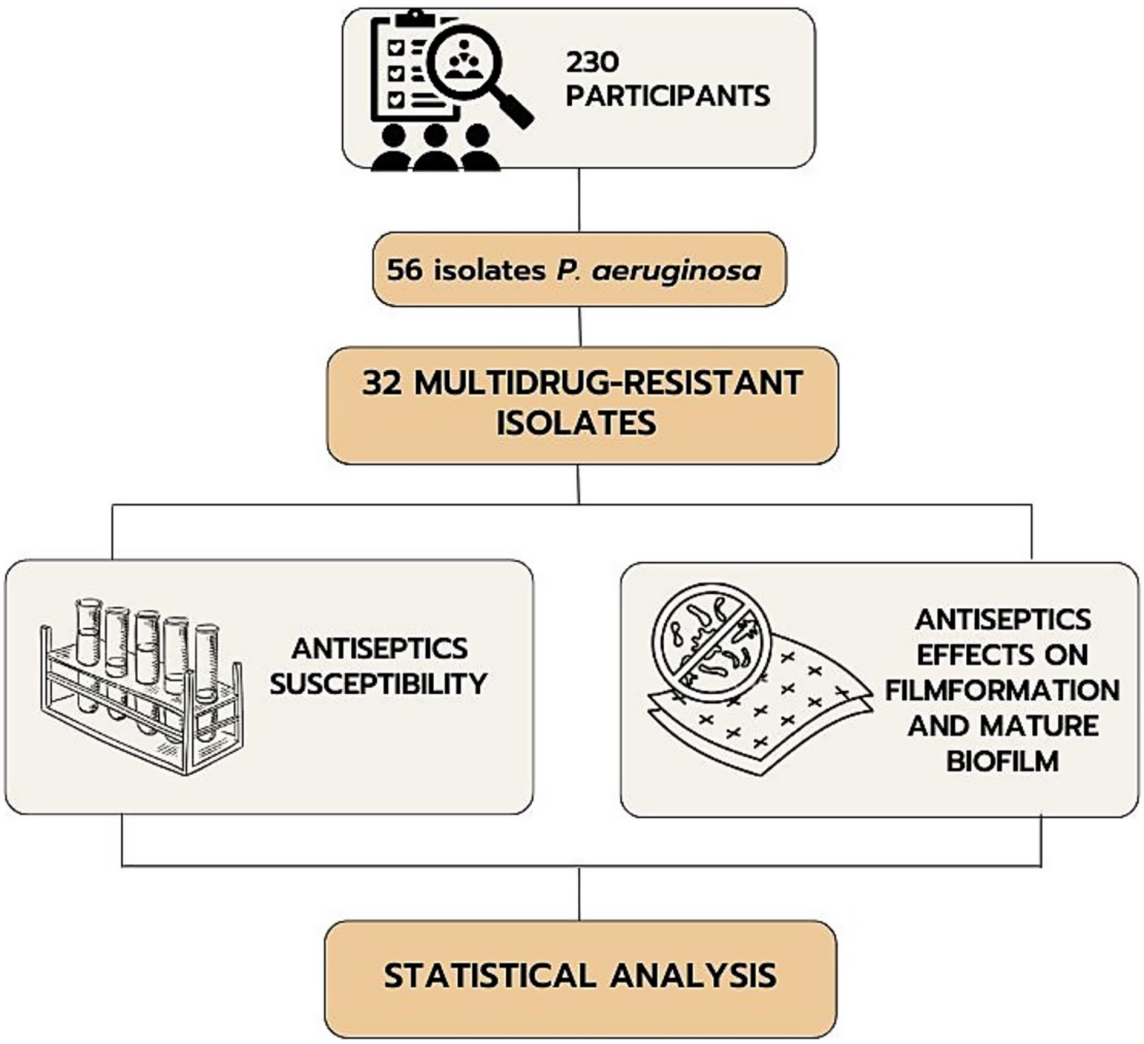
Flowchart of the study.

This cross-sectional study involved 230 patients with infected combat burns and shrapnel wounds of various localizations who were treated during 2022–2023. The inclusion criteria were the presence of infected combat wounds and the patient’s consent to participate in the study. The exclusion criteria were inconsistency of the diagnosis with the study objective, lack of consciousness in the patient, diabetes mellitus, congenital or acquired immunodeficiencies, mental disorders, and refusal to participate in the study. The study included the selection of multidrug-resistant *P. aeruginosa* among isolates from patients with the following determination of the sensitivity of their planktonic and film forms to antiseptics (Figure 1).

The material was obtained from the surface of burn or infected wounds using a sterile probe swab into transport tubes with Aimes medium, followed by cultivation under aerobic conditions at 37 °С. The final identification of clinical bacterial isolates was carried out by a standard bacteriological method, considering morphological, tentorial, cultural and biochemical properties of microorganisms using MIKRO-LA-Test kits (Erba Lachema, the Czech Republic).

The cohort included all patients with combined injuries of soft tissues, burns from who clinical isolates of gram-negative bacteria had been obtained. These patients were admitted from 12 different medical institutions of Ukraine (in 2022–2023) to provide them with specialized tertiary medical care. *Pseudomonas aeruginosa* (*n* = 56) was identified from patients with combined injuries.

In total 56 clinical isolates of *P. aeruginosa* were tested for antibiotic susceptibility to select MDR representatives. There were 9 antimicrobial agents from 6 antimicrobial categories used to characterize the resistance profile of *P. aeruginosa* isolates using the disk diffusion method (Kirby-Bauer test) according to EUCAST recommendations (Version 14.0, valid from 2024-01-01). MDR isolates were classified based on the criteria defined by Magiorakos et al. (2012). A total of 32 MDR strains were selected based on their resistance to one or more agents in three or more categories indicating the MDR category. The reference bacterial strain *Pseudomonas aeruginosa ATCC 27853* from the American Type Culture Collection (Manassas, Virginia, USA) was used as a control.

### 2.2 Determination of susceptibility of MDR bacteria to antiseptics

The susceptibility tests of clinical MDR and reference *P. aeruginosa* strains were performed by the standard method of double serial dilutions in Mueller-Hinton broth (HiMedia Laboratories, India) according to the recommendations of ISO standard 20,776–1:2019 (CLSI, USA). Daily bacterial cultures were resuspended with a final concentration of 5 × 10^5^ CFUs/ml (McFarland 0.5). Consecutive two-fold dilutions of the antiseptics were prepared. Then 0.1 mL of bacterial suspension was added to each tube and incubated for 24 h at 37 °C. The determination of minimum inhibitory concentration (MIC) and minimum bactericidal concentration (MBC) by antiseptics was carried after inoculation of the contents of the tubes on Mueller-Hinton agar.

The obtained values of MIC and MBC were also recorded as the bacteriostatic index of antiseptic activity (BS IAA) as the ratio of the initial concentration of the antiseptic to its MIC, and the bactericidal index of antiseptic activity (BC IAA) as the ratio of the initial concentration of the antiseptic to its MBC, respectively. The antiseptic was considered active (bacteriostatic and bactericidal activity, respectively) if the IAA value was greater than four (IAA > 4), since under natural conditions the effectiveness of the antiseptic decreases 4-fold (Denysko et al., 2022). Information on the antiseptics included in the study is provided in Table 1.

**Table 1:** Antiseptics tested.

Product (country of manufacturer)	Abbreviation	Active ingredient	Initial concentration (%)	Initial concentration (μg/ml)
Octenidine (Germany)	OCT	octenidine dihydrochloride	0.1	1,000
Polyhexanide (Germany)	PHMB	polyhexamethylene biguanide	0.1	1,000
Chlorhexidine (Ukraine)	CHG	chlorhexidine digluconate	0.5	500
Miramistin (Ukraine)	MRM	miramistin	0.01	100
Decamethoxine (Ukraine)	DCM	decamethoxine	0.1	1,000
Decasan (Ukraine)	DCM	decamethoxine	0.02	200
Povidone-iodine (Hungary)	PVP-I	povidone-iodine	10 2 (dil. 1:5) 1 (dil. 1:10)	100,00020,000 10,000

### 2.3 Determination of the effect of antiseptics on immature and mature biofilms

The ability of the studied strains to form a biofilm was modeled using the microtiter plate method with sterile 96-well flat-bottomed polystyrene trays. The inhibition of biofilm formation was assessed by introducing an antiseptic in subbacteriostatic concentrations into the well along with the bacterial culture. The destruction of mature biofilm was studied under the influence of bacteriostatic, bactericidal, and half of the initial concentration of antiseptics.

A daily culture of bacteria in planktonic form, suspended in tryptic soy broth (TSB, EMD Millipore, Burlington, Massachusetts, USA) with 1% glucose, with a concentration of ~10^5^ CFUs/ml, which corresponds to McFarland 0.5, was used. The negative control wells were inoculated with culture medium.

To simulate the inhibition of biofilm formation, 100 μL of the prepared suspension and 100 μL of the antiseptic solution at a concentration of 2 ˟ 1/2 MIC were added to a sterile 96-well flat-bottomed microtitration plate (USA Scientific), reaching a final antiseptic concentration of 1/2 MIC in the well. The plates were cultured in a humidified chamber in a thermostat at 37 °C for 24 h. After incubation, planktonic cells were removed from the wells by pipetting, the plate was washed three times with phosphate-buffered saline (PBS), pH 7.2 (Sigma, USA; cat. no. P-3813), fixed with Bouin solution, and stained with 150 μL of 2.0% crystal violet (Hucker formulation) for 15 min at room temperature. Thereafter, the optical density of the solution was measured at 620 nm. The intensity of staining of the well contents is directly proportional to the degree of biofilm formation; the quantitative expression of biofilm formation activity is the value of optical density, which was measured on a STAT FAX®4,300 spectrophotometer (the Netherlands) and expressed in optical density units (ODU). The value of ODU < 0.120 was evaluated as a low ability to form biofilms, 0.221–0.239 - as average, ODU > 0.240 - as a high indicator.

To determine the ability of antiseptics at MIC, MBC and ½ of the original concentration to destroy mature biofilm, planktonic cells were removed from the plate with the tested strain cultures after 72 h of incubation and 100 μL of antiseptic solution at concentrations of 2 ˟ MIC, 2 ˟ 1/2 MBC and at the original concentration were added to each well to achieve the tested concentrations. The incubation was then continued for 24 h in a humid chamber in a thermostat at 37 °C. The further procedure was similar to the one outlined above.

### 2.4 Statistical analysis

The mean, standard deviation, median, minimum, maximum frequency, and percentage were used for descriptive statistics. Student’s *t*-test was used to compare two normally distributed groups. One-way analysis of variance (ANOVA: one factor) was used to compare the results of three or more groups of data. The Bonferroni correction adjusted the significance level to control for the overall probability of errors (false positives) for testing multiple hypotheses. The result was considered reliable if the *p*-value was less than 0.05. Statistical data processing was performed using licensed Microsoft Office (365) Excel 2019, IBM SPSS Statistics version 22.0, and GraphPad Prism Software 10.1.0 (US, 2023).

## 3 Results

### 3.1 Antiseptic susceptibility testing

The MIC and MBC values of most antiseptics against *P. aeruginosa* strains were consistently lower than their initial commercial concentrations. Antiseptics from the detergent group as exemplified by decamethoxine (0.1 and 0.02%) and polyhexanide (0.1%) demonstrated the highest antimicrobial activity. As can be seen from the data in Table 2, the bacteriostatic concentrations of these antiseptics were 63.2 ± 5.2 μg/mL and 68.7 ± 4.2 μg/mL, respectively, and the bactericidal concentrations were 107.9 ± 5.8 μg/mL and 103.2 ± 12.9 μg/mL. The mean values of MIC for miramistin, chlorhexidine and octenidine were 94.2 ± 2.5 μg/mL, 95.8 ± 13.2 μg/mL and 84.7 ± 7.6 μg/mL, respectively. The MBC values for chlorhexidine and octenidine were 193.9 ± 22.9 μg/mL and 155.5 ± 16.4 μg/mL. As for the antiseptic Miramistin, the initial concentration of the active substance of this agent (100 μg/mL) was not sufficient to determine the bactericidal concentration, i.e., the MIC values against *P. aeruginosa* strains were > 100 μg/mL.

**Table 2:** Characteristics of the bacteriostatic and bactericidal effects of antiseptics on *Pseudomonas aeruginosa* strains (*n* = 33), in μg/ml (arithmetic mean ± arithmetic mean error: M ± m).

Antiseptics	MІC	MBC	0.5 MIC	BC ІАА/ BS ІАА
Octenidine	84.7 ± 7.6	155.5 ± 16.4	42.3 ± 3.8	0.5
Polyhexanide	68.7 ± 4.2	103.2 ± 12.9	35.6 ± 2.1	0.9
Chlorhexidine	95.8 ± 13.3	193.9 ± 22.90	47.9 ± 6.6	0.6
Miramistin	94.2 ± 2.5	>100	47.1 ± 1.2	-
Decamethoxine	63.2 ± 5.2	107.9 ± 5.9	30.9 ± 2.7	0.5
Decasan	60.2 ± 5.1	107.0 ± 9.3	30.09 ± 2.5	0.6
Povidone-iodine	3314.0 ± 369.5	5552.3 ± 682.6	1657.0 ± 184.7	0.6

A comparison of the data and an assessment of the reliability of their differences showed that polyhexanide was 1.37-fold more effective than miramistin in inhibiting the growth of *P. aeruginosa* (*p* < 0.001). Decamethoxine inhibited the growth of MDR *P. aeruginosa* strains 1.49-fold more effectively than miramistin (*p* < 0.001), 1.51-fold more effectively than chlorhexidine (*p* < 0.05), and 1.34-fold more effectively than octenidine (*p* < 0.05). The bactericidal activity of polyhexanide was 1.59-fold higher than that of chlorhexidine (*p* < 0.01) and 1.51-fold higher than that of octenidine (*p* < 0.05). The bactericidal concentrations of decamethoxine were 1.8-fold lower than those of chlorhexidine (*p* < 0.001) and 1.44-fold lower than those of octenidine (*p* < 0.01).

Thus, the ranking of the effectiveness of antiseptic drugs by bacteriostatic properties was (from the most effective drug):

decamethoxine > polyhexanide > octenidine > miramistin > chlorhexidine.

The scale of bactericidal activity of drugs will be as follows (from the most active):

decamethoxine > polyhexanide > octenidine > chlorhexidine > miramistin.

Povidone-iodine, as an active substance from the halogen group, acts at significantly higher concentrations than antiseptics from the detergent group. Therefore, we cannot compare their bacteriostatic and bactericidal concentration values, but we can compare their activity. The MIC of povidone-iodine was 3313.95 ± 369.45 μg/mL, and the MIC of MBC was 5552.33 ± 682.63 μg/mL.

**Figure 2:**
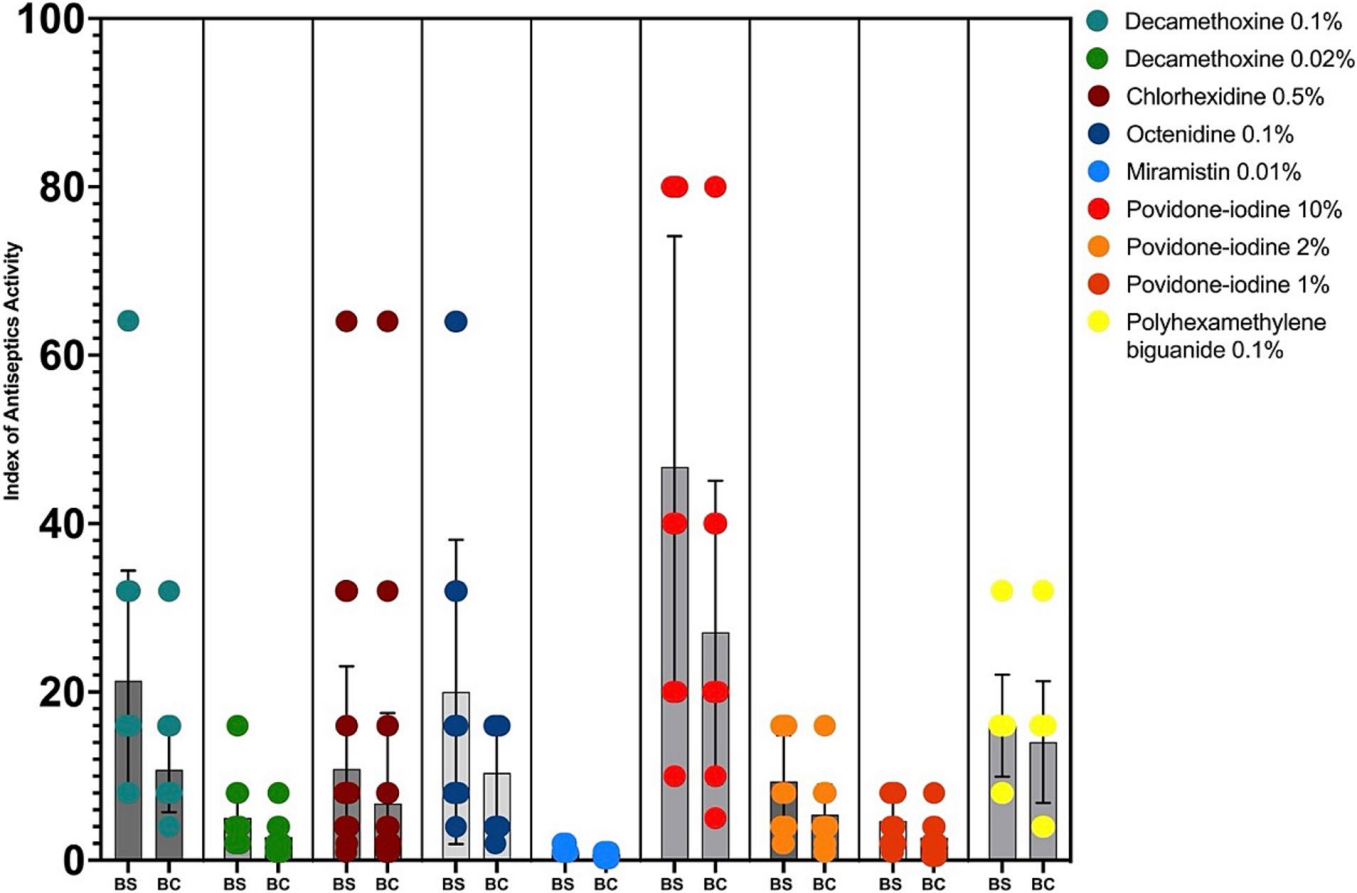
Bacteriostatic and bactericidal indexes of antiseptics activity against MDR *P. aeruginosa* (*n* = 32). BS – bacteriostatic, BC – bactericidal.

The interpretation of the results was also presented in the calculations of the bacteriostatic and bactericidal index of antiseptic activity (BS IAA and BC IAA) and their ratio (Figure 2; Table 2).

The antiseptic activity indices allow comparing drugs with different initial concentrations of active substance in the product, drugs of different chemical groups with different mechanisms of action, which makes it possible to assess the feasibility of using this drug and this particular concentration of active substance against a particular microorganism. “Active antiseptic” is characterized by an index of ≥4.

Since the Betadine® drug instruction (initial concentration of povidone-iodine - 10%) also recommends using a dilution of 1:5 and 1:10, the indices of antiseptic activity were additionally calculated for concentrations of povidone-iodine 2 and 1%.

The highest values of bacteriostatic IAA for clinical strains of *P. aeruginosa* with multidrug resistance were calculated for povidone iodine 10% (BS IAA = 46.7), decamethoxine 0.1% (BS IAA = 21.34), octenidine 0.1% (BS IAA = 20.0), polyhexanide 0.1% (BS IAA = 16.0). For decamethoxin 0.02%, the BS IAA was 5.02, for chlorhexidine 0.5% - 10.86, for povidone iodine 2% - 9.35, and for povidone iodine 1% - 4.67. The BS IAA of miramistin 0.01% was below the limit value and amounted to - 1.12. This concentration of the agent is not sufficient for use against MDR strains of *P. aeruginosa.*

The bactericidal activity indices took the highest values for povidone-iodine 10% (BC IAA = 27.09), decamethoxine 0.1% (BC IAA = 10.76), octenidine 0.1% (BC IAA = 10.37), polyhexanide 0.1% (BC IAA = 14.05). BC IAA of chlorhexidine 0.5% was 6.74, povidone-iodine 2% - 5.42. The bactericidal activity of decamethoxin 0.02% and povidone-iodine 1% was characterized by indices 2.77 and 2.71. The BC IAA of miramistin was not calculated, since the initial concentration of the agent was not sufficient to determine the bactericidal concentrations.

The highest cidal activity was found for polyhexanide: the ratio of BC IAA to BS IAA was 0.88. The values of the ratio BC IAA / BS IAA for all other antiseptics ranged from 0.5 to 0.6.

### 3.2 The effect of antiseptics on immature and formed biofilm of MDR strains of *P. aeruginosa*

The next step was to determine the sensitivity of biofilm forms of wound isolates of *P. aeruginosa* to antiseptics active against planktonic forms of these strains. The study showed that miramistin at its initial concentration of 0.01% had no activity against the planktonic forms of the studied bacteria (IAA ≤ 4), so this antiseptic was excluded from the biofilm testing.

All strains tested had the ability to form biofilms. Moreover, this property was interpreted as high, since the optical density values exceeded >0.240 units (average ODU = 0.415 ± 0.017).

#### 3.2.1 The effect of antiseptics on immature biofilm: efficiency of inhibition of biofilm formation

**Figure 3:**
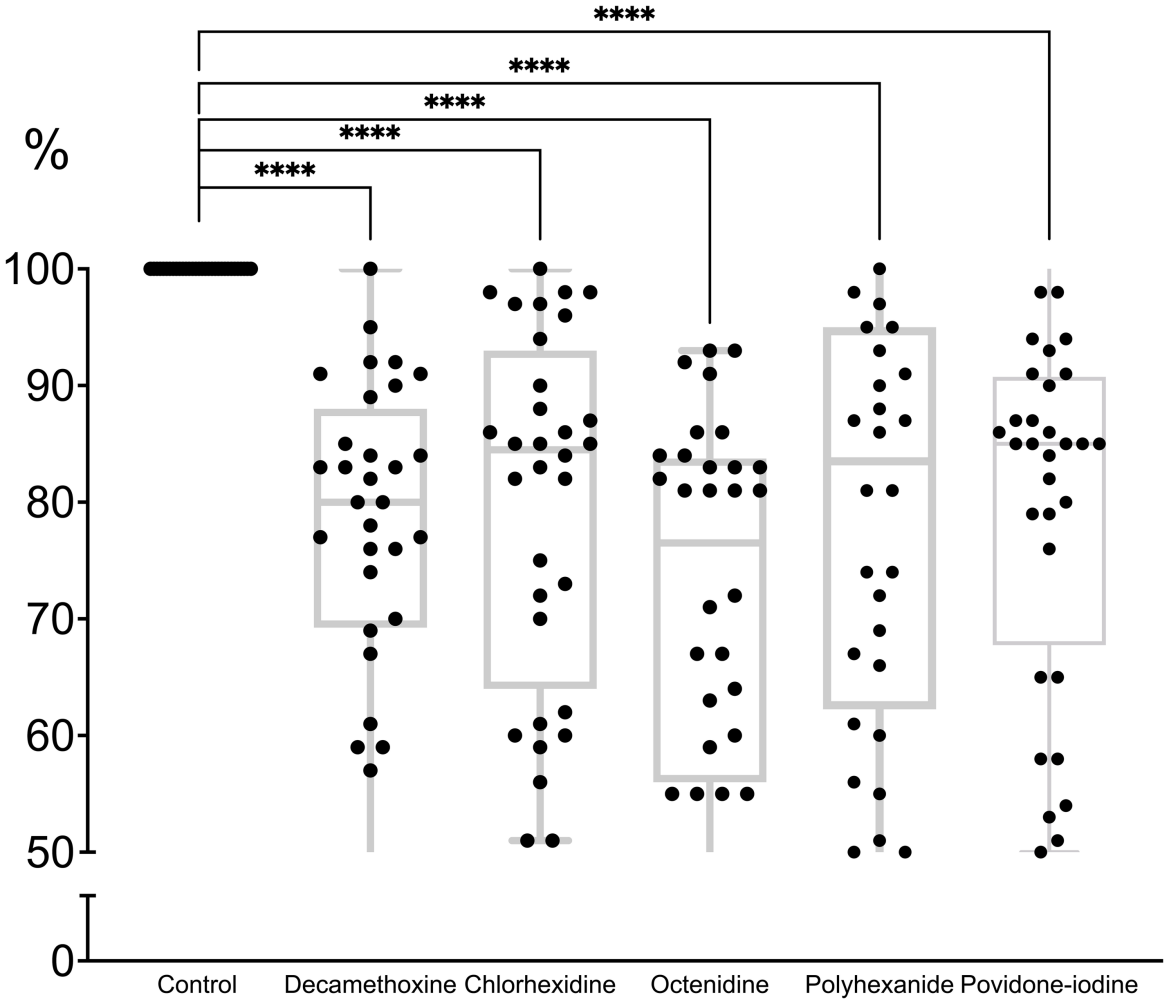
The effect of subbacteriostatic (1/2 MIC) antiseptic concentrations on biofilm formation by MDR *P. aeruginosa* strains (*n* = 32).

All antiseptics significantly inhibited biofilm formation (*p* < 0.0001). The percentage of inhibitory effect was 79.3 ± 2.6% for polyhexanide, 71.5 ± 2.8% for OCT, 79.8 ± 2.7% for chlorhexidine, 77.6 ± 2.4% for decamethoxine and 79.9 ± 2.6% for povidone-iodine compared to the control (100.0%). Octenidine in sub-MIC concentrations demonstrated the strongest effect on immature biofilm and inhibited its formation by 28.5% (*p* < 0.0001). Next on the scale of effectiveness were decamethoxin and polyhexanide, which significantly inhibited biofilm formation by 22.4 and 20.7% compared to the control (*p* < 0.0001). Chlorhexidine and povidone-iodine inhibited biofilm formation by 20.2 and 20.1%, respectively (Figure 3; Supplementary Table 1).

If we rank the effectiveness of drugs by the effect of their subbacteriostatic concentrations on the immature biofilm of multidrug-resistant pseudomonas, the scale of effectiveness will be as follows (from the most effective): octenidine > decamethoxine > polyhexanide > chlorhexidine > povidone iodine.

Octenidine showed the greatest activity against biofilm formation. As can be seen from Table 2, the bacteriostatic concentrations of octenidine are quite high, exceeding those of, for example, polyhexanide and decamethoxine. Thus, for the tested antiseptics from the group of detergents and halogen-containing compounds, the ability to inhibit biofilm formation depended on the concentration of the antiseptic, and not on the sensitivity of *P. aeruginosa* isolates to them.

#### 3.2.2 The effectiveness of antiseptics on preformed *P. aeruginosa* biofilm: evaluation of the effect of MIC, MBC and ½ of the initial concentration of antiseptics on the formed biofilm

**Figure 4:**
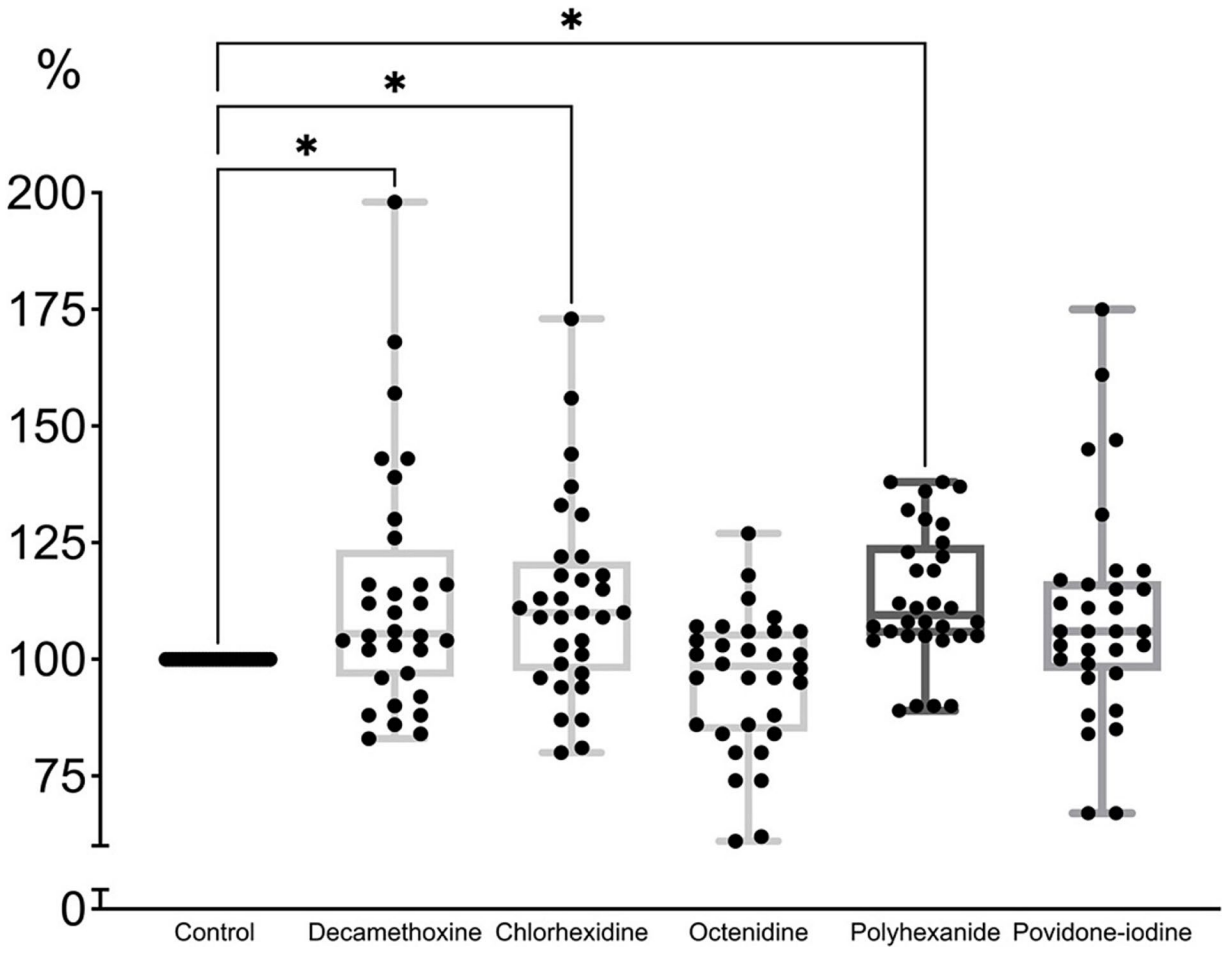
The effect of minimal inhibitory concentration (MIC) of antiseptics on the mature biofilm of *P. aeruginosa* (*n* = 32) in comparison with untreated control culture (in %).

As can be seen in Figure 4, the minimum bacteriostatic concentration of most antiseptics stimulated the protective forces of the biofilm as a form of organization approaching the tissue level. “Quorum sensing” ensured the reaction of the structure to a greater extent in the form of production of a protective matrix (Shree et al., 2023).

MIC of povidone-iodine stimulated the development of biofilm by 9.4%, bacteriostatic concentration of polyhexanide - by 13.2%, chlorhexidine - by 12.2%, decamethoxin - by 13.7% (*p* < 0.001). The bacteriostatic concentration of octenidine (average 84.67 ± 7.63 μg/mL) led to the eradication of biofilm by 4.7% (*p* < 0.001) compared to the control (Figure 4; Supplementary Table 2).

**Figure 5:**
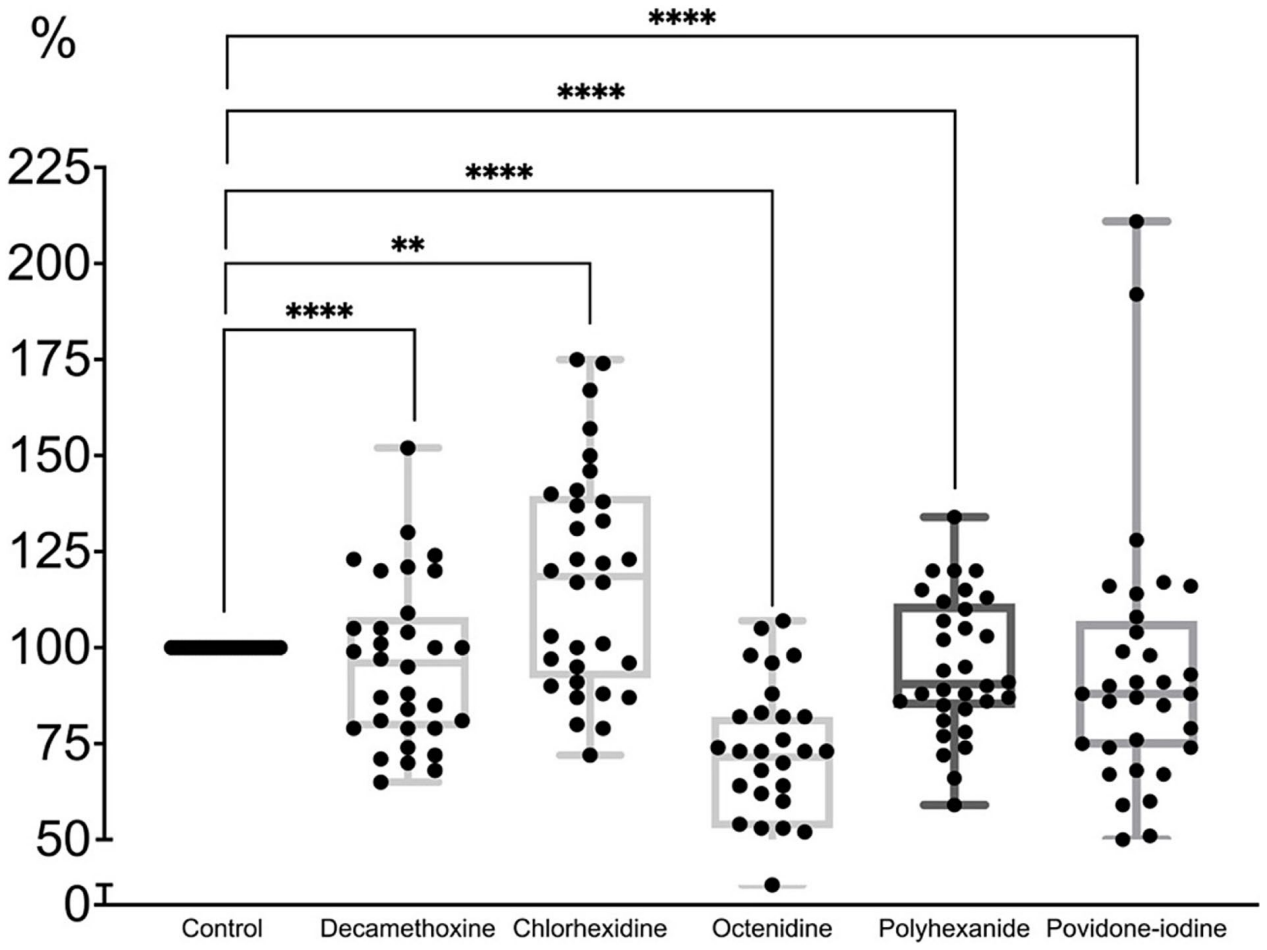
The effect of minimal bactericidal concentration of antiseptics on the mature biofilm of *P. aeruginosa* (*n* = 32) in comparison with untreated control culture (in %).

When most MBCs of antiseptics were applied to the formed MDR biofilm of *P. aeruginosa* strains (Figure 5; Supplementary Table 3), the latter was eradicated by 4% with decamethoxin (*p* < 0.001), by 4.8% with polyhexanide (*p* < 0.001), by 6.2% with povidone iodine (*p* < 0.001) and by 30.6% with octenidine (*p* < 0.001). Chlorhexidine stimulated the biofilm by 17.9% (*p* < 0.001).

Thus, the percentage of biofilm in comparison with the control was 93.8% under povidone-iodine, 95.2% under polyhexanide, 69.4% under octenidine, 117.9% under chlorhexidine, 96.0% under decamethoxine (*p* < 0.001).

**Figure 6:**
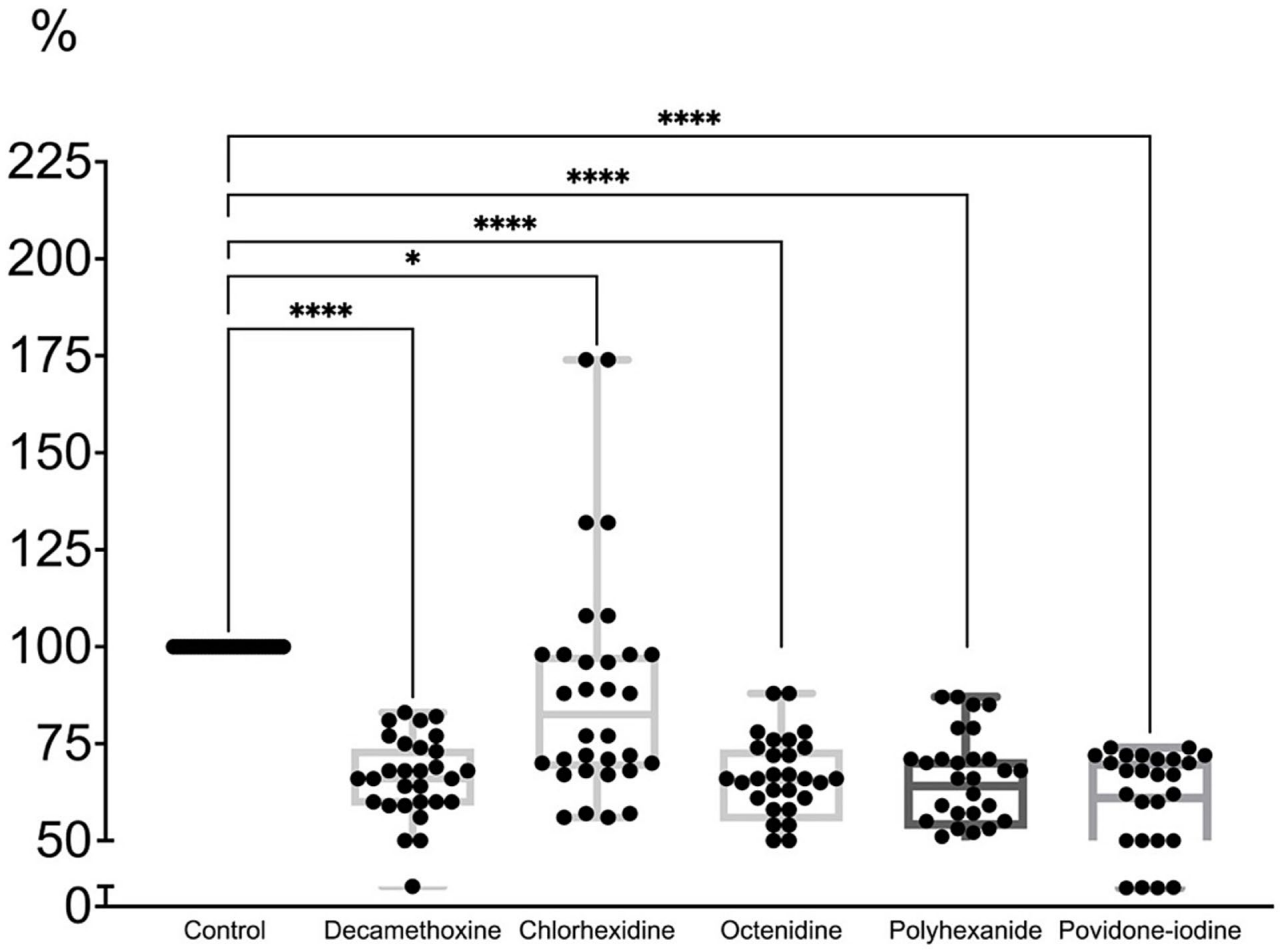
The effect of half of the initial concentration of antiseptics on the mature biofilm of *P. aeruginosa* (*n* = 32) compared to the untreated control (in %).

All antiseptics in a concentration equal to half the initial concentration of the active substance led to partial eradication of the MDR biofilm of pseudomonas strains by 11.3–42.4% (Figure 6; Supplementary Table 4).

The percentage of biofilm compared to the control under the action of decamethoxine was lower by 35.4% (*p* < 0.001) and amounted to 64.6%, under the action of chlorhexidine - by 11.3% (*p* < 0.001) and amounted to 88. 7%, under the influence of octenidine - by 35.8% (*p* < 0.001) and amounted to 64.2%, under the influence of polyhexanide - by 36.5% (*p* < 0.001) and amounted to 63.5%, under the influence of povidone-iodine - by 42.4% (*p* < 0.001) and amounted to 57.6%. Chlorhexidine showed the lowest activity against the formed biofilm at a concentration of half the initial concentration. However, its initial concentration is half that of other detergents.

Thus, the sensitivity to antiseptics of cultures in mature biofilms is much lower. An effective effect on the formed biofilm requires much higher concentrations of antiseptics. It is much easier to inhibit or prevent its formation. The tested concentrations of antiseptics do not destroy the formed biofilm by more than 42.4%. The ability of the antiseptics to eradicate the biofilm depended on the concentration: the highest tested concentrations were the most effective, equal to half the concentration of the finished commercial product.

Tracing the trend of octenidine action at different concentrations at different stages of biofilm formation, it should be noted that it is most active against *P. aeruginosa* biofilm (Supplementary Table 5).

## 4 Discussion

The emergence of multidrug resistance in bacteria has become one of the most dauntingchallenges of this century: the prevalence of infections that are difficult to treat is increasing, and there are no appropriate therapeutic alternatives. The scale of the problem has been identified by the political leaders of the G7 countries, who have expressed strong support for the first World Health Organization (WHO) Global Action Plan on Antimicrobial Resistance (AMR) [Rossi Gonçalves et al., 2017; Global Antibiotic Research and Development Partnership (GARDP), n.d.]. The global collaborative organization Joint Programming Initiative on Antimicrobial Resistance (JPIAMR) has engaged 29 countries in the fight against antimicrobial resistance, based on the One Health approach (JPIAMR, n.d.; CDC, 2019).

Infections caused by antibiotic-resistant *P. aeruginosa* are spreading steadily around the world due to its high internal resistance and ability to rapidly acquire resistance to all classes of antibiotics. The emergence of a specific resistance type of *P. aeruginosa*, namely the emergence of carbapenem-resistant (CRPA) strains, has attracted considerable attention from clinical microbiologists and infection control specialists (Moradali et al., 2017; Nolan and Behrends, 2021; Rossi Gonçalves et al., 2017; Rossolini et al., 2014; Oliver et al., 2015; Kovalchuk et al., 2024). The WHO recognizes carbapenem-resistant *P. aeruginosa* (CRPA) as a high priority pathogen for which antibiotic development is urgently needed (World Health Organization, 2024; Gergova et al., 2024). The emergence, spread, and persistence of multidrug-resistant bacteria, or “superbugs,” threatens human, animal, and environmental health as interconnected components of a single ecosystem (Davies and Davies, 2010; Aslam et al., 2021). *P. aeruginosa* MDR exists in a triangle-reservoir of animals, humans, and the environment, and there is interconnected coexistence of these pathogens within this triad. Numerous causes of “global resistance” contribute to the pressure of genetic selection and the emergence of bacterial MDR infections in society (CDC, 2019; Gergova et al., 2024; Van Boeckel et al., 2015; Aslam et al., 2018; Ahmad I et al., 2021; Crone et al., 2019; Balcázar et al., 2015; Abd El-Ghany, 2021).

*P. aeruginosa* is striking in the variety of pathology it causes, being the cause of a wide range of diseases - from intoxication to extensive purulent inflammatory processes and septic shock. Purulent complications of wound processes are very significant. In the general structure of wound infections, the *P. aeruginosa* bacterium occupies a significant place, being one of the most common bacteria (Ruffin and Brochiero, 2019; Wolcott et al., 2016; Phan et al., 2023). *Pseudomonas aeruginosa* infections in the epithelium of the skin, cornea and respiratory tract are the main cause of hospitalizations, disability and deaths worldwide (Ruffin and Brochiero, 2019). Along with *S. aureus, K. pneumoniae, E. coli, and Acinetobacter* spp., *P. aeruginosa* is among the leading superbugs that complicate the course of combat trauma (Mende et al., 2022; Weintrob et al., 2018; Petersen et al., 2007; Ford et al., 2017; Kvasnevska et al., 2024). One of the predictors of high mortality *P. aeruginosa* infections is multidrug resistance of the causative strain (Ruffin and Brochiero, 2019; Oliver et al., 2015), and infections caused by antibiotic-resistant *P. aeruginosa* are increasing worldwide (Nolan and Behrends, 2021; Rossolini et al., 2014; Oliver et al., 2015; Galdino et al., 2019; Pogue et al., 2020; Buehrle et al., 2016; Rubio et al., 2021; Kyaw et al., 2015). For example, Mareș, C. and colleagues reported an increase in antibiotic resistance in opportunistic pathogens due to the COVID-19 pandemic, and some of the highest rates of increase were observed for *P. aeruginosa* (Mareș et al., 2022).

The problem of *P. aeruginosa* infections requires a joint international interdisciplinary effort to translate current knowledge into strategies to prevent and treat *P. aeruginosa* infections, while reducing antibiotic resistance and avoiding the spread of resistant strains in nature, as patient sanitation is one of the key measures in efforts to break the epidemic chain by acting on the source of infection, thus preventing the spread of MDR strains (Moradali et al., 2017; Sanya et al., 2023; Theuretzbacher et al., 2020; Pang et al., 2019; Brüggemann et al., 2018; Rossi Gonçalves et al., 2017; Loban’ et al., 2023; Babalska et al., 2021). As correctly summarized by Kramer, A. and colleagues, wound antisepsis has experienced a renaissance due to the development of effective wound-compatible antiseptic agents, their bactericidal effect instead of bacteriostatic, the relatively high level of sensitization to topically applied antibiotics, also due to the pandemic spread of multidrug-resistant microorganisms, and, to the advantage, the absence (rarely) of resistance to those antiseptics that irreversibly damage pathogens (Kramer et al., 2018).

There are no generally accepted recommendations for the use of antiseptics for wounds. Regular monitoring (control) of sensitivity, correction of initial antiseptic concentrations with adjustment for multidrug-resistant strains of pathogens, and especially given the potential presence of such a widespread and resistant pathogen as pseudomonas are important and necessary. Suppression of the associated microflora with prolonged use of antibiotics sometimes leads to the fact that *P. aeruginosa* remains the only bacterial species in the infection site, impeding wound healing (Betchen et al., 2022; Kawamura et al., 2019). The results of our study indicate the high efficiency of modern antiseptics against MDR strains of *P. aeruginosa*. The MIC values of antiseptics (except for miramistin) against *P. aeruginosa* strains were always lower than the initial commercial concentrations. Certainly, the MBC for all microbicides were higher than their respective MICs, but the ratio of MBC/ MBS was less than 4, indicating that the products exhibit predominantly bactericidal properties (Betchen et al., 2022; Levison, 2004).

In recent studies by Barrigah-Benissan and colleagues, the MIC values for polyhexanide, povidone-iodine, and octenidine were also always lower than the original commercial concentrations (Barrigah-Benissan et al., 2022). Grzegorz Krasowski and colleagues determined the bactericidal concentrations of polyhexanide and octenidine at dilutions several tens of times below the threshold of the initial solution (Krasowski et al., 2021). Similarly, Rafael López-Rojas and colleagues previously found high activity of polyhexanide against clinical isolates of *P. aeruginosa* with the MDR phenotype at much lower concentrations than the initial ones (López-Rojas et al., 2017). Studies by Tomasz M. Karpiński characterized octenidine as a very effective drug against clinical wound isolates and reference strains of *P. aeruginosa*. However, for their sample of isolates, the MIC values for octenidine and polyhexanide did not differ from previous studies (López-Rojas et al., 2017; Karpiński, 2019; Koburger et al., 2010; Murray et al., 2015). We selected strains with the MDR phenotype, and the MIC and MIC values for the antiseptics studied were higher than they were in previous studies. The same trend, for example, was observed by Gupta, P. et al. for MDR of *P. aeruginosa* and povidone-iodine (Gupta et al., 2018). Vásquez, Daniel and colleagues also found high MICs and MBCs of chlohexidine in many home and hospital isolates of *P. aeruginosa* (Vásquez et al., *2017*). In our previous similar studies concerning other MDR opportunistic pathogens (Ljungquist et al., 2023; Kovalchuk et al., 2024; Nazarchuk et al., 2024), we referred to the review by Jean-Yves Maillard and colleagues, whose analysis convincingly confirmed the decrease in the sensitivity of wound pathogens, including *P. aeruginosa*, to all biocides, which is associated with the spread of resistance (Maillard et al., 2021). At the same time, antibiotics have more resistance determinants than antiseptics and disinfectants, and gene expression under the influence of antimicrobial agents is not a good predictor of these resistance determinants (Murray et al., 2015; Schultz et al., 2017). Antiseptics act on multiple targets, inside and on the surface of the bacterial cell, unlike antibiotics (Krasowski et al., 2021; Assadian, 2016). The antiseptic activity index allows you to assess the effectiveness and appropriateness of the drug, and compare antiseptics with each other. The antiseptic concentration should be at least 4 MIC. We interpreted the results using the differential IAA index, focusing on the cidal activity of the antiseptic (Kramer et al., 2018). According to the results of the evaluation of the activity of drugs based on bacteriostatic IAA, povidone-iodine 10%, decamethoxine 0.1%, octenidine 0.1%, polyhexanide 0.1%, decamethoxine 0.02%, chlorhexidine 0.5%, povidone-iodine 2%, povidone-iodine 1% are effective. The concentration of miramistin 0.01% is not sufficient for use against MDR strains of *P. aeruginosa*. The concentration of the active ingredient of this drug is the lowest among those studied here. According to the cidal activity index, the most effective are povidone-iodine 10%, decamethoxine 0.1%, octenidine 0.1%, polyhexanide 0.1% (BC IAA = 14.05), chlorhexidine 0.5%, povidone-iodine 2%. The highest values of the indices were taken for povidone-iodine 10%. The ratio of BC IAA to BS IAA in favor of cidal action was the highest for polyhexanide. Our research and that of colleagues from other countries shows that antiseptics, including those tested in this study, are effective against planktonic bacteria. However, pathogenic bacteria are mostly found in biofilms, as this is their natural state (Rossi Gonçalves et al., 2017; Günther et al., 2021; Nazarchuk et al., 2019).

Therefore, biofilm elimination is important from a therapeutic point of view and for infection control (Rossi Gonçalves et al., 2017; Maurice et al., 2018). An effective antiseptic used for the treatment of colonized/infected chronic wounds should exhibit biofilm control properties (Krasowski et al., 2021; Schultz et al., 2017). The data are not yet clear on whether the MDR phenotype correlates with biofilm-forming properties (Rossi Gonçalves et al., 2017). Some researchers have noted an increased ability to form biofilm by *P. aeruginosa* strains (Magiorakos et al., 2012; Kaiser et al., 2017; Karballaei Mirzahosseini et al., 2020; Gurung et al., 2013). In any case, the biofilm is an important factor in the virulence of *P. aeruginosa*, the main form of its existence, which protects against the harmful effects of environmental factors, including biocides, and also contributes to the persistence and spread of MDR strains (Yin et al., 2019; Uddin et al., 2021). This was not the aim of our study, but it should be noted that the *P. aeruginosa* strains tested by MDR were characterized by high biofilm-forming capacity. The same was observed by Rossi Gonçalves I. et al., Bakht, M. et al., Sanchez, C et al. Behzadi, P. et al., Cepas, V. et al. point out that, indeed, strong biofilm producers are more common among clinical isolates, but MDR status or resistance to individual antibiotics does not imply an increased ability to form biofilms (Rossi Gonçalves et al., 2017; Bakht et al., 2022). This selection is logical, since biofilm-forming strains survive better and have a better chance of acquiring the determinants of acquired resistance. However, these are most likely not genetically linked traits.

Our studies of the effect of antiseptics on immature biofilm, i.e., their effectiveness in inhibiting biofilm formation, showed that all antiseptics have a high level of inhibitory capacity. Octenidine in sub-MIC concentrations showed the strongest effect on immature biofilm. Decamethoxine, polyhexanide, chlorhexidine, and povidone-iodine were next on the scale of effectiveness. A negative correlation was found between the ability of MDR strains of *P. aeruginosa* to form biofilms in the presence of subbacteriostatic concentrations of antiseptics and the susceptibility of these isolates to antiseptics. Thus, for the tested antiseptics from the group of detergents and halogen-containing compounds, the ability to inhibit biofilm formation depended on the concentration of the antiseptic, not on the sensitivity of *P. aeruginosa* isolates to them.

The sensitivity to antiseptics of cultures in mature biofilms was much lower. An effective effect on the formed biofilm requires much higher concentrations of antiseptics. It is much easier to inhibit or prevent its formation. The tested concentrations of antiseptics do not destroy the formed biofilm by more than 42.4%. The ability of the antiseptics to eradicate the biofilm depended on the concentration: the highest tested concentrations were the most effective, equal to half the concentration of the finished commercial product. Tracing the trend of octenidine action at different concentrations at different stages of biofilm formation, it should be noted that it is most active against *P. aeruginosa* biofilm. But, in general, it should be noted that all tested antiseptics are effective against *P. aeruginosa* biofilm. Junka A et al. also noted the high activity of octenidine and povidone iodine against biofilms of nosocomial *P. aeruginosa* strains (Sanchez Jr et al., 2013; Cepas et al., 2019; Junka et al., 2014). The results obtained by Grzegorz Krasowski et al. also indicate a high anti-biofilm activity of antiseptics based on polyhexanide and octenidine. The researchers note that antiseptics based on polyhexanide or octenidine are very useful for treating biofilm (Levison, 2004). Gryson, L et al. recently studied the anti-biofilm activity of povidone iodine and polyhexnide and reported that PVP-I and PHMB demonstrated sustained activity against biofilms *in vitro*, and PVP-I led to complete eradication of 3- and 5-day-old *Pseudomonas aeruginosa* biofilms (in ≤0.5 h) (Gryson et al., 2023).

There have also been important advances in the development of strategies for treating infections caused by *P. aeruginosa* and the use of combination therapy. Elodie Lefebvre et al. used a combination of polyhexanide, EDTA, and proteases in low concentrations, which had a synergistic effect that led to the complete eradication of dense *P. aeruginosa* biofilms (Lefebvre et al., 2016). Ciecholewska-Juśko D investigated the phenomenon of increasing the activity of an octenidine dihydrochloride-based antiseptic against *Pseudomonas aeruginosa* biofilms in the presence of a rotating magnetic field of two frequencies of 5 and 50 Hz. The authors noted that the combination of a rotating magnetic field and OCT may be particularly promising for the destruction of biofilms located in areas such as wound pockets, where physical obstacles limit antiseptic activity (Ciecholewska-Juśko et al., 2022).

*P. aeruginosa* has an innate resistance to many classes of drugs, the ability to form biofilms and, most importantly, the ability to quickly acquire resistance after treatment. One of the obvious unfortunate consequences of increased resistance to antimicrobial drugs is that bacteria are often treated at concentrations below their minimum inhibitory concentration (Nolan and Behrends, 2021). For example, in terms of biocides in general, Daniel Vásquez and colleagues note that hospitals with highly resistant strains of *P. aeruginosa* and *A. baumannii* with high drug resistance, it is necessary to review new formulations in cleaning and disinfection protocols (Vásquez et al., 2017). Also, Rasha Gharieb and colleagues report that carbapenem-resistant *P. aeruginosa* (CRPA) on intensive livestock farms is a serious problem that threatens animal and human health and increases the risk of *P. aeruginosa* infection in the community, so it is vital to control the spread of CRPA by limiting the use of antibiotics and applying proper cleaning and disinfection protocols on livestock farms (Ciecholewska-Juśko et al., 2022; Rasha Gharieb et al., 2021).

Regular monitoring of susceptibility, development of new therapeutic strategies against multidrug-resistant *P. aeruginosa*, correction of initial antiseptic concentrations with adjustment for multidrug-resistant strains and bacterial bloom status are relevant and important. The potential presence of such a resistant pathogen as *P. aeruginosa* should always be taken into account. It should be treated and prevented by following the “One Health” strategy.

## 5 Conclusion

The most active antiseptics against *P. aeruginosa* MDR are decamethoxin 0.1%, polyhexanide 0.1%, octenidine 0.1%, povidone-iodine 10%. The efficacy of miramistin 0.01% was found to be insufficient, as the IAA was below the threshold value (<4). Octenidine in sub-MIC concentrations demonstrated the strongest effect on immature biofilm (on its formation). The minimum bacteriostatic concentration of most antiseptics stimulated the development of mature biofilm. The bacteriostatic concentration of octenidine led to the eradication of mature biofilm by 4.7%. The MBC of most antiseptics (except chlorhexidine) led to eradication of mature biofilm by 4–30.6%. Chlorhexidine stimulated mature biofilm by 17.9%. Chlorhexidine showed the lowest activity against the formed biofilm at a concentration of half the initial concentration. But its initial concentration is half that of other detergents. The tested concentrations of antiseptics do not destroy the formed biofilm by more than 42.4%.

Tracing the trend of octenidine action at different concentrations at different stages of biofilm formation, its highest activity against *P. aeruginosa* biofilm should be emphasized. The results indicate the possibility of wider use of octenidine and decamethoxin for treatment of surgery wounds in patients with infection caused by MDR *P. aeruginosa* with possible recommendation for inclusion in wound infection treatment protocols.

The results of our study emphasize the importance of careful monitoring of *P. aeruginosa* isolates for antiseptic susceptibility. This will ultimately help prevent the creation of selective conditions for the emergence of resistant microorganisms and prevent their spread.

## Data Availability

The raw data supporting the conclusions of this article will be made available by the authors, without undue reservation.
