# Supplementary material for: Modern antiseptics against multidrug-resistant Pseudomonas aeruginosa, emerging from war-related injuries in Ukraine
Source: Front Microbiol. 2025 Oct 31;16:1656270. doi: 10.3389/fmicb.2025.1656270 (PMC12615205; doi:10.3389/fmicb.2025.1656270)
Supplement: Supplementary file 1 [file Table_1.DOCX]

Supplementary Material

# Supplementary Table 1

**The effect of subbacteriostatic (1/2 MIC) antiseptic concentrations on biofilm formation by MDR *P. aeruginosa* strains (*n*=32)**

| Isolates | **Decamethoxine** | | **Chlorhexidine** | | **Octenidine** | | **Polyhexanide** | | **Povidone-iodine** | | **Control** | |
| --- | --- | --- | --- | --- | --- | --- | --- | --- | --- | --- | --- | --- |
|  | ODU | % | ODU | % | ODU | % | ODU | % | ODU | % | ODU | % |
|  | 0,315 | 61 | 0,323 | 62 | 0,285 | 55 | 0,291 | 56 | 0,304 | 58 | 0,520 | 100 |
|  | 0,362 | 59 | 0,310 | 51 | 0,280 | 46 | 0,312 | 51 | 0,312 | 51 | 0,609 | 100 |
|  | 0,367 | 83 | 0,323 | 73 | 0,262 | 59 | 0,291 | 66 | 0,333 | 76 | 0,441 | 100 |
|  | 0,323 | 83 | 0,339 | 87 | 0,261 | 67 | 0,314 | 81 | 0,332 | 85 | 0,390 | 100 |
|  | 0,351 | 67 | 0,314 | 60 | 0,289 | 55 | 0,311 | 60 | 0,337 | 65 | 0,521 | 100 |
|  | 0,296 | 48 | 0,348 | 56 | 0,277 | 45 | 0,304 | 49 | 0,328 | 53 | 0,618 | 100 |
|  | 0,320 | 74 | 0,366 | 85 | 0,310 | 72 | 0,321 | 74 | 0,368 | 85 | 0,433 | 100 |
|  | 0,433 | 100 | 0,311 | 72 | 0,273 | 63 | 0,291 | 67 | 0,342 | 79 | 0,433 | 100 |
|  | 0,308 | 59 | 0,31 | 60 | 0,286 | 55 | 0,286 | 55 | 0,302 | 58 | 0,520 | 100 |
|  | 0,349 | 57 | 0,313 | 51 | 0,270 | 44 | 0,305 | 50 | 0,304 | 50 | 0,609 | 100 |
|  | 0,351 | 80 | 0,332 | 75 | 0,266 | 60 | 0,304 | 69 | 0,351 | 80 | 0,441 | 100 |
|  | 0,328 | 84 | 0,351 | 90 | 0,261 | 67 | 0,314 | 81 | 0,332 | 85 | 0,390 | 100 |
|  | 0,359 | 69 | 0,32 | 61 | 0,286 | 55 | 0,319 | 61 | 0,34 | 65 | 0,521 | 100 |
|  | 0,298 | 48 | 0,363 | 59 | 0,280 | 45 | 0,311 | 50 | 0,336 | 54 | 0,618 | 100 |
|  | 0,328 | 76 | 0,365 | 84 | 0,309 | 71 | 0,319 | 74 | 0,369 | 85 | 0,433 | 100 |
|  | 0,305 | 70 | 0,305 | 70 | 0,275 | 64 | 0,311 | 72 | 0,341 | 79 | 0,433 | 100 |
|  | 0,258 | 92 | 0,272 | 97 | 0,228 | 81 | 0,273 | 97 | 0,245 | 87 | 0,281 | 100 |
|  | 0,232 | 83 | 0,262 | 94 | 0,229 | 82 | 0,274 | 98 | 0,240 | 86 | 0,279 | 100 |
|  | 0,229 | 89 | 0,249 | 97 | 0,234 | 91 | 0,262 | 102 | 0,251 | 98 | 0,256 | 100 |
|  | 0,257 | 91 | 0,239 | 85 | 0,233 | 83 | 0,262 | 93 | 0,256 | 91 | 0,282 | 100 |
|  | 0,225 | 77 | 0,252 | 86 | 0,236 | 81 | 0,258 | 88 | 0,254 | 87 | 0,292 | 100 |
|  | 0,223 | 76 | 0,245 | 83 | 0,253 | 86 | 0,256 | 87 | 0,241 | 82 | 0,294 | 100 |
|  | 0,251 | 95 | 0,257 | 98 | 0,244 | 93 | 0,272 | 103 | 0,246 | 94 | 0,263 | 100 |
|  | 0,239 | 82 | 0,238 | 82 | 0,244 | 84 | 0,251 | 86 | 0,248 | 85 | 0,291 | 100 |
|  | 0,259 | 92 | 0,282 | 100 | 0,228 | 81 | 0,281 | 100 | 0,253 | 90 | 0,281 | 100 |
|  | 0,237 | 85 | 0,267 | 96 | 0,232 | 83 | 0,266 | 95 | 0,253 | 91 | 0,279 | 100 |
|  | 0,231 | 90 | 0,250 | 98 | 0,235 | 92 | 0,266 | 104 | 0,261 | 102 | 0,256 | 100 |
|  | 0,256 | 91 | 0,241 | 85 | 0,234 | 83 | 0,269 | 95 | 0,261 | 93 | 0,282 | 100 |
|  | 0,227 | 78 | 0,256 | 88 | 0,237 | 81 | 0,264 | 90 | 0,275 | 94 | 0,292 | 100 |
|  | 0,225 | 77 | 0,253 | 86 | 0,252 | 86 | 0,267 | 91 | 0,247 | 84 | 0,294 | 100 |
|  | 0,221 | 84 | 0,259 | 98 | 0,244 | 93 | 0,274 | 104 | 0,257 | 98 | 0,263 | 100 |
|  | 0,234 | 80 | 0,239 | 82 | 0,244 | 84 | 0,253 | 87 | 0,251 | 86 | 0,291 | 100 |

Notes: ODU – optical density units.

# 2. Supplementary Table 2

**The effect of minimal inhibitory concentration (MIC) of antiseptics on the mature biofilm of *P. aeruginosa* (*n*=32) in comparison with untreated control culture (in %).**

| Isolates | **Decamethoxine** | | **Chlorhexidine** | | **Octenidine** | | **Polyhexanide** | | **Povidone-iodine** | | **Control** | |
| --- | --- | --- | --- | --- | --- | --- | --- | --- | --- | --- | --- | --- |
|  | ODU | % | ODU | % | ODU | % | ODU | % | ODU | % | ODU | % |
|  | 0,254 | 88 | 0,278 | 97 | 0,230 | 80 | 0,260 | 90 | 0,254 | 88 | 0,288 | 100 |
|  | 0,278 | 116 | 0,280 | 117 | 0,253 | 106 | 0,284 | 119 | 0,265 | 111 | 0,239 | 100 |
|  | 0,275 | 104 | 0,267 | 101 | 0,253 | 96 | 0,277 | 105 | 0,281 | 106 | 0,264 | 100 |
|  | 0,277 | 112 | 0,270 | 109 | 0,251 | 102 | 0,325 | 132 | 0,294 | 119 | 0,247 | 100 |
|  | 0,276 | 96 | 0,271 | 94 | 0,241 | 84 | 0,300 | 104 | 0,276 | 96 | 0,288 | 100 |
|  | 0,278 | 116 | 0,270 | 113 | 0,256 | 107 | 0,256 | 107 | 0,275 | 115 | 0,239 | 100 |
|  | 0,281 | 106 | 0,274 | 104 | 0,254 | 96 | 0,285 | 108 | 0,272 | 103 | 0,264 | 100 |
|  | 0,272 | 110 | 0,272 | 110 | 0,249 | 101 | 0,277 | 112 | 0,283 | 115 | 0,247 | 100 |
|  | 0,252 | 88 | 0,275 | 96 | 0,229 | 80 | 0,257 | 90 | 0,254 | 89 | 0,285 | 100 |
|  | 0,271 | 114 | 0,272 | 115 | 0,252 | 106 | 0,283 | 119 | 0,262 | 111 | 0,237 | 100 |
|  | 0,275 | 105 | 0,260 | 99 | 0,249 | 95 | 0,273 | 104 | 0,280 | 106 | 0,263 | 100 |
|  | 0,276 | 112 | 0,267 | 109 | 0,249 | 101 | 0,321 | 130 | 0,288 | 117 | 0,246 | 100 |
|  | 0,276 | 97 | 0,269 | 94 | 0,239 | 84 | 0,299 | 105 | 0,276 | 97 | 0,285 | 100 |
|  | 0,276 | 116 | 0,267 | 113 | 0,253 | 107 | 0,255 | 108 | 0,275 | 116 | 0,237 | 100 |
|  | 0,276 | 105 | 0,270 | 103 | 0,252 | 96 | 0,284 | 108 | 0,268 | 102 | 0,263 | 100 |
|  | 0,257 | 104 | 0,268 | 109 | 0,249 | 101 | 0,275 | 112 | 0,275 | 112 | 0,246 | 100 |
|  | 0,333 | 84 | 0,321 | 81 | 0,246 | 62 | 0,358 | 90 | 0,268 | 67 | 0,398 | 100 |
|  | 0,343 | 102 | 0,290 | 87 | 0,249 | 74 | 0,459 | 137 | 0,285 | 85 | 0,335 | 100 |
|  | 0,451 | 143 | 0,384 | 122 | 0,278 | 88 | 0,349 | 111 | 0,333 | 106 | 0,315 | 100 |
|  | 0,343 | 130 | 0,454 | 173 | 0,333 | 127 | 0,322 | 122 | 0,460 | 175 | 0,263 | 100 |
|  | 0,343 | 86 | 0,529 | 133 | 0,343 | 86 | 0,418 | 105 | 0,395 | 99 | 0,398 | 100 |
|  | 0,343 | 102 | 0,368 | 110 | 0,347 | 104 | 0,418 | 125 | 0,485 | 145 | 0,335 | 100 |
|  | 0,290 | 92 | 0,384 | 122 | 0,312 | 99 | 0,335 | 106 | 0,323 | 103 | 0,315 | 100 |
|  | 0,414 | 157 | 0,378 | 144 | 0,310 | 118 | 0,362 | 138 | 0,345 | 131 | 0,263 | 100 |
|  | 0,331 | 83 | 0,319 | 80 | 0,246 | 61 | 0,356 | 89 | 0,267 | 67 | 0,401 | 100 |
|  | 0,346 | 103 | 0,293 | 87 | 0,248 | 74 | 0,466 | 138 | 0,283 | 84 | 0,337 | 100 |
|  | 0,453 | 139 | 0,383 | 118 | 0,278 | 86 | 0,348 | 107 | 0,333 | 102 | 0,325 | 100 |
|  | 0,583 | 198 | 0,459 | 156 | 0,319 | 109 | 0,325 | 111 | 0,473 | 161 | 0,294 | 100 |
|  | 0,504 | 126 | 0,548 | 137 | 0,394 | 98 | 0,492 | 123 | 0,401 | 100 | 0,401 | 100 |
|  | 0,567 | 168 | 0,375 | 111 | 0,381 | 113 | 0,459 | 136 | 0,497 | 147 | 0,337 | 100 |
|  | 0,292 | 90 | 0,385 | 118 | 0,335 | 103 | 0,340 | 105 | 0,344 | 106 | 0,325 | 100 |
|  | 0,42 | 143 | 0,385 | 131 | 0,313 | 106 | 0,378 | 129 | 0,351 | 119 | 0,294 | 100 |

Notes: ODU – optical density units.

# 3. Supplementary Table 3

**The effect of minimal bactericidal concentration of antiseptics on the mature biofilm of *P. aeruginosa* (*n*=32) in comparison with untreated control culture (in %)**

| Isolates | **Decamethoxine** | | **Chlorhexidine** | | **Octenidine** | | **Polyhexanide** | | **Povidone-iodine** | | **Control** | |
| --- | --- | --- | --- | --- | --- | --- | --- | --- | --- | --- | --- | --- |
|  | ODU | % | ODU | % | ODU | % | ODU | % | ODU | % | ODU | % |
|  | 0,380 | 72 | 0,415 | 79 | 0,259 | 49 | 0,391 | 74 | 0,416 | 79 | 0,527 | 100 |
|  | 0,380 | 99 | 0,382 | 100 | 0,268 | 70 | 0,423 | 110 | 0,380 | 99 | 0,383 | 100 |
|  | 0,439 | 109 | 0,415 | 103 | 0,330 | 82 | 0,349 | 87 | 0,374 | 93 | 0,402 | 100 |
|  | 0,380 | 121 | 0,491 | 157 | 0,330 | 105 | 0,375 | 120 | 0,401 | 128 | 0,313 | 100 |
|  | 0,392 | 74 | 0,378 | 72 | 0,229 | 43 | 0,310 | 59 | 0,392 | 74 | 0,527 | 100 |
|  | 0,372 | 97 | 0,335 | 87 | 0,246 | 64 | 0,330 | 86 | 0,377 | 98 | 0,383 | 100 |
|  | 0,403 | 100 | 0,407 | 101 | 0,330 | 82 | 0,366 | 91 | 0,363 | 90 | 0,402 | 100 |
|  | 0,407 | 130 | 0,415 | 133 | 0,256 | 82 | 0,351 | 112 | 0,365 | 117 | 0,313 | 100 |
|  | 0,451 | 104 | 0,504 | 117 | 0,259 | 60 | 0,364 | 84 | 0,376 | 87 | 0,432 | 100 |
|  | 0,389 | 105 | 0,360 | 97 | 0,269 | 73 | 0,351 | 95 | 0,383 | 104 | 0,370 | 100 |
|  | 0,396 | 105 | 0,363 | 96 | 0,330 | 88 | 0,330 | 88 | 0,343 | 91 | 0,377 | 100 |
|  | 0,467 | 152 | 0,430 | 140 | 0,330 | 107 | 0,352 | 115 | 0,350 | 114 | 0,307 | 100 |
|  | 0,340 | 79 | 0,344 | 80 | 0,330 | 76 | 0,312 | 72 | 0,327 | 76 | 0,432 | 100 |
|  | 0,323 | 87 | 0,337 | 91 | 0,238 | 64 | 0,328 | 89 | 0,324 | 88 | 0,370 | 100 |
|  | 0,360 | 95 | 0,360 | 95 | 0,234 | 62 | 0,321 | 85 | 0,330 | 88 | 0,377 | 100 |
|  | 0,379 | 123 | 0,375 | 122 | 0,255 | 83 | 0,328 | 107 | 0,331 | 108 | 0,307 | 100 |
|  | 0,380 | 84 | 0,677 | 150 | 0,236 | 52 | 0,543 | 120 | 0,866 | 192 | 0,452 | 100 |
|  | 0,404 | 120 | 0,415 | 123 | 0,330 | 98 | 0,291 | 86 | 0,391 | 116 | 0,337 | 100 |
|  | 0,365 | 65 | 0,687 | 123 | 0,222 | 40 | 0,369 | 66 | 0,338 | 60 | 0,560 | 100 |
|  | 0,380 | 70 | 0,740 | 137 | 0,221 | 41 | 0,477 | 88 | 0,271 | 50 | 0,542 | 100 |
|  | 0,357 | 79 | 0,637 | 141 | 0,330 | 73 | 0,512 | 113 | 0,304 | 67 | 0,452 | 100 |
|  | 0,298 | 88 | 0,587 | 174 | 0,330 | 98 | 0,355 | 105 | 0,291 | 86 | 0,337 | 100 |
|  | 0,381 | 68 | 0,503 | 90 | 0,229 | 41 | 0,576 | 103 | 0,288 | 51 | 0,560 | 100 |
|  | 0,386 | 71 | 0,648 | 120 | 0,227 | 42 | 0,421 | 78 | 0,369 | 68 | 0,542 | 100 |
|  | 0,41 | 120 | 0,599 | 175 | 0,233 | 68 | 0,411 | 120 | 0,723 | 211 | 0,343 | 100 |
|  | 0,374 | 124 | 0,504 | 167 | 0,220 | 73 | 0,285 | 94 | 0,351 | 116 | 0,302 | 100 |
|  | 0,337 | 81 | 0,607 | 146 | 0,222 | 53 | 0,321 | 77 | 0,31 | 74 | 0,417 | 100 |
|  | 0,380 | 85 | 0,521 | 117 | 0,330 | 74 | 0,401 | 90 | 0,265 | 59 | 0,447 | 100 |
|  | 0,346 | 101 | 0,448 | 131 | 0,330 | 96 | 0,458 | 134 | 0,29 | 85 | 0,343 | 100 |
|  | 0,301 | 100 | 0,417 | 138 | 0,220 | 73 | 0,308 | 102 | 0,276 | 91 | 0,302 | 100 |
|  | 0,338 | 81 | 0,363 | 87 | 0,224 | 54 | 0,479 | 115 | 0,278 | 67 | 0,417 | 100 |
|  | 0,352 | 79 | 0,393 | 88 | 0,236 | 53 | 0,363 | 81 | 0,337 | 75 | 0,447 | 100 |

Notes: ODU – optical density units.

# 4. Supplementary Table 4

**The effect of half of the initial concentration of antiseptics on the mature biofilm of *P. aeruginosa* (*n*=32) compared to the untreated control (in %)**

| Isolates | **Decamethoxine** | | **Chlorhexidine** | | **Octenidine** | | **Polyhexanide** | | **Povidone-iodine** | | **Control** | |
| --- | --- | --- | --- | --- | --- | --- | --- | --- | --- | --- | --- | --- |
|  | ODU | % | ODU | % | ODU | % | ODU | % | ODU | % | ODU | % |
|  | 0,258 | 49 | 0,293 | 56 | 0,249 | 47 | 0,226 | 43 | 0,215 | 41 | 0,527 | 100 |
|  | 0,252 | 66 | 0,267 | 70 | 0,241 | 63 | 0,262 | 68 | 0,275 | 72 | 0,383 | 100 |
|  | 0,257 | 64 | 0,268 | 67 | 0,247 | 61 | 0,267 | 66 | 0,272 | 68 | 0,402 | 100 |
|  | 0,252 | 81 | 0,301 | 96 | 0,239 | 76 | 0,267 | 85 | 0,218 | 70 | 0,313 | 100 |
|  | 0,258 | 49 | 0,293 | 56 | 0,249 | 47 | 0,267 | 51 | 0,215 | 41 | 0,527 | 100 |
|  | 0,252 | 66 | 0,267 | 70 | 0,241 | 63 | 0,262 | 68 | 0,275 | 72 | 0,383 | 100 |
|  | 0,257 | 64 | 0,268 | 67 | 0,247 | 61 | 0,267 | 66 | 0,272 | 68 | 0,402 | 100 |
|  | 0,252 | 81 | 0,301 | 96 | 0,239 | 76 | 0,267 | 85 | 0,218 | 70 | 0,313 | 100 |
|  | 0,258 | 60 | 0,293 | 68 | 0,249 | 58 | 0,267 | 62 | 0,215 | 50 | 0,432 | 100 |
|  | 0,252 | 68 | 0,267 | 72 | 0,241 | 65 | 0,262 | 71 | 0,275 | 74 | 0,370 | 100 |
|  | 0,257 | 68 | 0,268 | 71 | 0,247 | 66 | 0,224 | 59 | 0,272 | 72 | 0,377 | 100 |
|  | 0,224 | 73 | 0,301 | 98 | 0,239 | 78 | 0,267 | 87 | 0,218 | 71 | 0,307 | 100 |
|  | 0,258 | 60 | 0,293 | 68 | 0,249 | 58 | 0,226 | 52 | 0,215 | 50 | 0,432 | 100 |
|  | 0,252 | 68 | 0,267 | 72 | 0,241 | 65 | 0,262 | 71 | 0,275 | 74 | 0,370 | 100 |
|  | 0,257 | 68 | 0,268 | 71 | 0,247 | 66 | 0,224 | 59 | 0,272 | 72 | 0,377 | 100 |
|  | 0,252 | 82 | 0,301 | 98 | 0,239 | 78 | 0,267 | 87 | 0,218 | 71 | 0,307 | 100 |
|  | 0,265 | 59 | 0,597 | 132 | 0,302 | 67 | 0,241 | 53 | 0,211 | 47 | 0,452 | 100 |
|  | 0,252 | 75 | 0,296 | 88 | 0,224 | 66 | 0,239 | 71 | 0,201 | 60 | 0,337 | 100 |
|  | 0,252 | 45 | 0,321 | 57 | 0,300 | 54 | 0,267 | 48 | 0,209 | 37 | 0,560 | 100 |
|  | 0,269 | 50 | 0,481 | 89 | 0,224 | 41 | 0,245 | 45 | 0,204 | 38 | 0,542 | 100 |
|  | 0,265 | 59 | 0,597 | 132 | 0,302 | 67 | 0,241 | 53 | 0,211 | 47 | 0,452 | 100 |
|  | 0,224 | 66 | 0,296 | 88 | 0,224 | 66 | 0,239 | 71 | 0,201 | 60 | 0,337 | 100 |
|  | 0,224 | 40 | 0,321 | 57 | 0,300 | 54 | 0,237 | 42 | 0,209 | 37 | 0,560 | 100 |
|  | 0,269 | 50 | 0,481 | 89 | 0,224 | 41 | 0,245 | 45 | 0,204 | 38 | 0,542 | 100 |
|  | 0,265 | 77 | 0,597 | 174 | 0,302 | 88 | 0,241 | 70 | 0,211 | 62 | 0,343 | 100 |
|  | 0,252 | 83 | 0,296 | 98 | 0,224 | 74 | 0,239 | 79 | 0,201 | 67 | 0,302 | 100 |
|  | 0,287 | 69 | 0,321 | 77 | 0,300 | 72 | 0,237 | 57 | 0,209 | 50 | 0,417 | 100 |
|  | 0,269 | 60 | 0,481 | 108 | 0,224 | 50 | 0,245 | 55 | 0,204 | 46 | 0,447 | 100 |
|  | 0,265 | 77 | 0,597 | 174 | 0,302 | 88 | 0,241 | 70 | 0,211 | 62 | 0,343 | 100 |
|  | 0,224 | 74 | 0,296 | 98 | 0,224 | 74 | 0,239 | 79 | 0,201 | 67 | 0,302 | 100 |
|  | 0,252 | 60 | 0,321 | 77 | 0,300 | 72 | 0,237 | 57 | 0,209 | 50 | 0,417 | 100 |
|  | 0,252 | 56 | 0,481 | 108 | 0,224 | 50 | 0,245 | 55 | 0,204 | 46 | 0,447 | 100 |

Notes: ODU – optical density units.

# 4. Supplementary Table 5

**Summary of the effect of antiseptics MIC on the filmformation and mature biofilm of MDR *P. aeruginosa* (*n*=32) compared to the untreated control (in %)**

| Isolates | PTZ | CAZ | CID | IMI | MER | CIP | GEN | TOB | COL | Decamethoxine | | Chlorhexidine | | Octenidine | | Polyhexanide | | Povidone-iodine | |
| --- | --- | --- | --- | --- | --- | --- | --- | --- | --- | --- | --- | --- | --- | --- | --- | --- | --- | --- | --- |
|  |  |  |  |  |  |  |  |  |  | Filmformation, % | Mature, % | Filmformation, % | Mature, % | Filmformation, % | Mature, % | Filmformation, % | Mature, % | Filmformation, % | Mature, % |
| 1 | I | R | S | R | S | R | R | R | S | 61 | 88 | 62 | 97 | 55 | 80 | 56 | 90 | 58 | 88 |
| 2 | I | I | R | R | S | R | R | S | R | 59 | 116 | 51 | 117 | 46 | 106 | 51 | 119 | 51 | 111 |
| 3 | I | I | S | R | S | R | R | S | R | 83 | 104 | 73 | 101 | 59 | 96 | 66 | 105 | 76 | 106 |
| 4 | R | R | S | R | S | R | S | S | S | 83 | 112 | 87 | 109 | 67 | 102 | 81 | 132 | 85 | 119 |
| 5 | R | R | S | R | S | I | S | S | R | 67 | 96 | 60 | 94 | 55 | 84 | 60 | 104 | 65 | 96 |
| 6 | R | R | S | R | S | R | S | S | S | 48 | 116 | 56 | 113 | 45 | 107 | 49 | 107 | 53 | 115 |
| 7 | I | I | R | I | R | I | R | S | S | 74 | 106 | 85 | 104 | 72 | 96 | 74 | 108 | 85 | 103 |
| 8 | I | I | R | R | I | R | R | S | S | 100 | 110 | 72 | 110 | 63 | 101 | 67 | 112 | 79 | 115 |
| 9 | R | R | R | I | I | R | R | R | S | 59 | 88 | 60 | 96 | 55 | 80 | 55 | 90 | 58 | 89 |
| 10 | I | I | R | R | I | R | S | S | S | 57 | 114 | 51 | 115 | 44 | 106 | 50 | 119 | 50 | 111 |
| 11 | R | R | R | R | R | R | S | R | S | 80 | 105 | 75 | 99 | 60 | 95 | 69 | 104 | 80 | 106 |
| 12 | R | R | S | R | R | R | S | S | S | 84 | 112 | 90 | 109 | 67 | 101 | 81 | 130 | 85 | 117 |
| 13 | I | R | S | R | R | R | R | S | S | 69 | 97 | 61 | 94 | 55 | 84 | 61 | 105 | 65 | 97 |
| 14 | R | R | S | R | R | R | R | R | S | 48 | 116 | 59 | 113 | 45 | 107 | 50 | 108 | 54 | 116 |
| 15 | R | R | S | R | R | R | R | R | S | 76 | 105 | 84 | 103 | 71 | 96 | 74 | 108 | 85 | 102 |
| 16 | R | R | S | R | R | R | R | R | S | 70 | 104 | 70 | 109 | 64 | 101 | 72 | 112 | 79 | 112 |
| 17 | R | R | S | R | R | R | R | R | S | 92 | 84 | 97 | 81 | 81 | 62 | 97 | 90 | 87 | 67 |
| 18 | R | R | S | R | R | R | R | R | R | 83 | 102 | 94 | 87 | 82 | 74 | 98 | 137 | 86 | 85 |
| 19 | R | R | R | R | R | R | R | R | R | 89 | 143 | 97 | 122 | 91 | 88 | 102 | 111 | 98 | 106 |
| 20 | R | R | S | R | R | R | R | R | R | 91 | 130 | 85 | 173 | 83 | 127 | 93 | 122 | 91 | 175 |
| 21 | R | R | S | R | R | R | R | R | R | 77 | 86 | 86 | 133 | 81 | 86 | 88 | 105 | 87 | 99 |
| 22 | R | R | S | R | R | R | R | R | R | 76 | 102 | 83 | 110 | 86 | 104 | 87 | 125 | 82 | 145 |
| 23 | R | R | S | R | R | R | R | R | R | 95 | 92 | 98 | 122 | 93 | 99 | 103 | 106 | 94 | 103 |
| 24 | R | R | R | R | R | R | R | R | R | 82 | 157 | 82 | 144 | 84 | 118 | 86 | 138 | 85 | 131 |
| 25 | S | R | R | R | S | R | R | S | R | 92 | 83 | 100 | 80 | 81 | 61 | 100 | 89 | 90 | 67 |
| 26 | R | R | R | I | I | I | R | R | S | 85 | 103 | 96 | 87 | 83 | 74 | 95 | 138 | 91 | 84 |
| 27 | S | R | R | R | I | R | R | S | R | 90 | 139 | 98 | 118 | 92 | 86 | 104 | 107 | 102 | 102 |
| 28 | S | R | R | R | I | R | R | S | R | 91 | 198 | 85 | 156 | 83 | 109 | 95 | 111 | 93 | 161 |
| 29 | S | R | S | R | R | R | R | R | R | 78 | 126 | 88 | 137 | 81 | 98 | 90 | 123 | 94 | 100 |
| 30 | S | R | S | R | R | R | R | R | R | 77 | 168 | 86 | 111 | 86 | 113 | 91 | 136 | 84 | 147 |
| 31 | S | R | S | R | R | R | R | R | R | 84 | 90 | 98 | 118 | 93 | 103 | 104 | 105 | 98 | 106 |
| 32 | S | R | S | R | R | R | R | R | R | 80 | 143 | 82 | 131 | 84 | 106 | 87 | 129 | 86 | 119 |

Note: PTZ- Piperacillin/tazobactam, CAZ- Ceftazidime, CID- Cefiderocol, IMI- Imipenem, MER- Meropenem, CIP- Ciprofloxacin, GEN- Gentamicin, TOB- Tobramycin, COL- Colistin
